# Dr. A. F. Sheldon

**Published:** 1914-02

**Authors:** 


Dr. A. F. Sheldon, N. Y. University 1852, died at his home in Lyons, January 4, aged 84. He served in the Civil War as surgeon to the 7th N. Y. Cavalry, 78th N. Y. Infantry, and as an executive officer on Gen. Wadsworth's staff in the Medical Director's Office at Washington. He was breveted Lt. Col. by President Johnson. He was well known as a practitioner of Wayne County, and had held many positions of honor in public and military capacities.

